# Is there a relationship between Eustachian tube dysfunction and nasal septal deviation in a sample of the Lebanese population?

**DOI:** 10.1186/s13005-020-00238-y

**Published:** 2020-10-06

**Authors:** Victoria Al Karaki, Souheil Hallit, Mansour Nacouzi, Ziad Rohayem

**Affiliations:** 1 Faculty of Medicine and Medical Sciences, Holy Spirit University of Kaslik (USEK), Jounieh, Lebanon; 2 INSPECT-LB: Institut National de Santé Publique, Épidémiologie Clinique et Toxicologie-Liban, Beirut, Lebanon; 3 Department of otorhinolaryngology, Eye and Ear Hospital, Naccache, Lebanon

## Abstract

**Background:**

The Eustachian tube (ET) is considered an organ by itself due to its specific functions. An ET Dysfunction (ETD) is discussed when this tube is unable to ventilate the middle ear properly. Clinically, the patient reports usually some aural fullness, “popping”, “under water” sensation as if the ear is clogged. This condition is common affecting at least 5% of the adult population. It can impair quality of life and become disabling. On the other side, the prevalence of nasal septal deviation (NSD) is believed to be around 22.83% in the adult population. Nasal septal deviation is thought to cause a decline in the middle ear ventilation according to certain authors. The primary outcome is to define the predictive value of the side of Eustachian Tube Dysfunction (ETD) symptoms vis-à-vis the side of nasal septal deviation (NSD) in patients having the two conditions concomitantly.

**Methods:**

A cross-sectional study was conducted between July 2018 and September 2019. Overall, 60 consecutive subjects (total of 120 ears), randomly seen at the Otorhinolaryngology Outpatient Clinics at the Eye and Ear International Hospital, Lebanon, all year-round were enrolled and tested without any geographic preferences. The Eustachian Tube Dysfunction Questionnaire (ETDQ) -7 questionnaire was used to evaluate ETD.

**Results:**

A significantly higher ETD score was found in males compared to females, in those with left septal deviation compared to right and in those who have symptoms on the left compared to right side. Frequent exposure to higher changes in altitude (commute from home to workplace) was also significantly associated with higher ETD scores (r = 0.265), whereas higher Left Tympanometric peak pressure (TPP in daPa) on tympanometry was significantly associated with lower ETD score (r = − 0.467). Furthermore, 25 patients who had symptoms on the left side had also a septal deviation to the left side (86.2%), whereas 29 (93.5%) patients who had the symptoms on the right side had septal deviation to the right side (*p* < 0.001).

**Conclusion:**

Our data highlighted the importance of altitude and geographic distribution of patients especially in a population exposed to barotrauma on a daily basis like the Lebanese population. Tympanometry, on the other hand, failed to correlate with patient reported symptoms and thus needs further evaluation. The reported ETD symptoms of the patient correlates to the side of NSD.

## Background

The Eustachian tube (ET) is considered an organ by itself due to its specific functions [1]. Anatomically, the tube’s length is 37 mm, settling in the petrous bone of the temporal lobe, extending from the anterior wall of the middle ear to the rhinopharynx [2]. It is divided into the cartilaginous part (medial two thirds) and the osseous part (external one third) [2]. As for its functions, starting with pressure equalization and ventilation of the middle ear, this tube opens its ostium to balance the air in the nasopharynx and secondly permits middle ear mucosal gas exchange [3]. In addition, this organ executes a muscular peristaltic motion allowing clearance of a great part of the middle ear secretions enhancing the activity of the mucociliary escalator and thus protecting against any infectious or inflammatory process [1]. An ET dysfunction is discussed when this tube is unable to perform any of these functions, reflecting clinically a trouble in the middle ear ventilation [1].

In this study we focus on obstructive ET dysfunction which is by definition failure to open and ventilate the middle ear and not patulous ET dysfunction known to be failure of ET closure [4]. The patient reports usually some particular symptoms including aural fullness, “popping”, “under water” sensation as if the ear is clogged, with occasionally tinnitus, imbalance, or even pain [1]. Although few epidemiologic studies have looked at the prevalence of ETD, this condition is believed to be nevertheless fairly common affecting at least 5% of the adult population [3, 5]. When it becomes chronic (lasting more than 3 months), it can impair quality of life and become disabling. In its most severe forms, it can even lead to hearing impairment [6]. Communication troubles, social withdrawal, altered motivation and engagement are subsequently seen [5]. More specifically, otologic impairment can range from tympanic membrane retractions, to adhesions, recurrent ear infections or even potentially cause cholesteatomas.

Tubomanometry was introduced in Europe in 2003 as a diagnostic tool for ETD, but there is until this date no consensus in the medical literature regarding its usefulness [7]. As such, it is not widely used in studies on ETD. For Sobegi et al., a C tympanogram reflects an ET dysfunction, which is a frequent pattern of subclinical middle ear malfunctions [8]. Although a negative middle ear tube pressure on tympanometry can indicate an ET dysfunction, the diagnosis remains a clinical one in the vast majority of cases. Based on this, McCoul et al. suggested in 2012 the Eustachian Tube Dysfunction Questionnaire (ETDQ) -7, which was consequently validated to include patient reported symptoms and to assess them objectively [3, 9].

The nasal septum is a cartilaginous structure that plays a major role in supporting and shaping the nose [10, 11]. It separates the two nasal fossae, thus dividing the intranasal airflow passage in two. Division is commonly asymmetric [11]. It’s the most common entity responsible of severe and chronic nose blockage [12, 13]. Estimated prevalence of nasal septal deviation is believed to be around 22.83% in the adult population [11]. Nasal obstruction compromises sinus drainage and alters the airflow passageways and resistance [11]. Many etiologies has been discussed for nasal septal deviation: trauma, growth, ossification and congenital malformations. Anterior rhinoscopy and nasal endoscopy are usually the only necessary exams for evaluating the nasal septum.

Although nasal septal deviation (NSD) is fairly common, it is relatively poorly discussed in the medical literature and therefore lacks any validated measuring instrument to quantitatively assess it. Regarding middle ear pressure, NSD is thought to cause a decline in the middle ear ventilation according to certain authors. To the best of our knowledge, no association has been established yet between the side of the reported symptoms of ET dysfunction and the side of nasal septal deviation. There is therefore a need for well-conducted studies looking at the relation between the two entities. Lebanon has very peculiar geography being a mountainous country with a very narrow coastal strip along the sea. Population is often subjected to large altitude changes even while commuting from home to work. This daily exposure to extreme changes in altitude makes our population a very distinctive one when it comes to interpreting the ETDQ-7 validated scale.

The primary aim of this study is to define the predictive value of the side of ET dysfunction symptoms vis-à-vis the side of nasal septal deviation in patients having the two conditions concomitantly.

## Methods

### Study design

A cross-sectional, study was conducted between July 2018 and September 2019. Overall, 60 consecutive subjects (total of 120 ears), randomly seen at the Otorhinolaryngology Outpatient Clinics at the Eye and Ear International Hospital, Lebanon, all year-round were enrolled and tested without any geographic preferences. Prior to participation, patients who reported symptoms of ETD dysfunction were given a briefing about the study and they all had the right to accept or refuse involvement. The age limit was set to be above 18 years to avoid any Eustachian tube growth adverse issues commonly seen in the pediatric population. The exclusion criteria were: History of cancer or radiotherapy to the Head and Neck region, craniofacial anomalies, any history of nasal or ear surgery, recent upper respiratory tract infection or recurrent ear infections, evidence of eardrum perforation on otoscopy and finally history of sleep apnea and the use of a CPAP machine.

### Procedures

Data was compiled through interviewer-administered questionnaires. The investigators who did the interviews were blind to the study. The time needed to complete the questionnaire was limited between 10 and 12 min and the interviewer respected the Arabic native language in Lebanon. The anonymity of the participants was assured. First, the questionnaire was translated into Arabic by a certified translator and then translated back to English by another translator. After the pre-test of equivalence by bilingual translators, the translated Arabic version was used and the recruitment phase started. It covered three sections.

#### Section a

assessed the socio-demographics details of the patient: age, gender and altitude. Exposure to barotrauma is very common in the Lebanese population regarding the fact that the mountains are 30 min away from the coast by car. Numerous citizens are prone to change in ear pressure with altitude, starting from zero meters on the coast and reaching rapidly 800–1000 m within 30–45 min. The XXXXX international is located on the coast in XXXXX at an altitude of 50 m from sea level and offers care to patients coming from various parts of Lebanon.

#### Section B

included information related to the medical background and habits of the patient: weight, allergy, obstructive sleep apnea (OSA), reported symptoms and their side, smoking, recurrent head and neck infections, cancer and previous surgeries. Symptoms were assessed through ETDQ7- questionnaire for ETD, as well as the validated Score for Allergic Rhinitis (SFAR) for allergies or a previous documented skin allergy testing and medication history. History of OSA was based on C-PAP usage or history of previous polysomnogram and the remainder information was gathered through yes or no questions. Patients were asked first about the side of the symptoms and then assessed for their complaints through the questionnaire. Assessing the side of deviation came next, only patients with one-sided deviation were kept for the study and then we documented if it’s right or left, for data to be analyzed concluding if it is the same side of the reported symptoms.

### The Eustachian tube dysfunction questionnaire (ETDQ) -7 questionnaire

The ETDQ-7 questionnaire comprises 7 items with a scale of graduated responses ranging from ‘1’ to ‘7’ allowing quantitative measurement of subjective complaints. Starting from ‘1’ corresponding to ‘no symptoms’ and proceeding gradually till ‘7’ indicating the maximum severity of the discomfort [2]. It includes 7 components: (1) pressure in the ears; (2) pain in the ears; (3) feeling the ears are under water; (4) ear symptoms when the patient has a cold or sinusitis; (5) cracking or popping sounds; (6) ringing in the ears; (7) feeling that hearing is muffled [12]. A score of less than 14.5 was considered normal [14].

### Score for allergic rhinitis (SFAR)

The SFAR is a 9-items allergic symptom score used to recognize patients with allergic rhinitis [15]. Each question targets a specific symptom: (1) nasal symptoms in the past year (blocked nose, runny nose, sneezing) 1 point for each symptom; (2) rhinoconjonctivitis; (3) season or months of the year; (4) pollens, house dust mites, dust; (5) cats and dogs); (6) previous allergic status; (7) previous positive allergy tests; (8) previous medical diagnosis of allergy; (9) familial history of allergy [15]. It is a quantitative measurement ranging between ‘0’ and ‘16’. A score above 7 indicates the presence of allergic rhinitis [15].

#### Section C

registered the clinical examination, otoscopy, nasal endoscopy and tympanometry. A diligent clinical examination was carried out especially to the Head and Neck region.

### Otoscopy

Otoscopy is used to screen for ear pathologies in case of discomfort. In our study, it was helpful in excluding cerumen impaction, otitis media, abnormal eardrum (e.g. retraction) or perforation. A pneumatic otoscopy by insufflating air to check for tympanic movements to rule out effusion was also performed.

### Nasal endoscopy

A nasal rigid endoscopy with a 4 mm 0 degree rod-lens endoscope was systematically performed to examine the nasal cavities and exclude sinusitis, polyps, and tumors. However, most importantly, it was the gold standard to evaluate presence of a nasal septal deviation and assess the side of the deviation. Definition of nasal septal deviation was based on symptoms and physical exam. Patients were questioned about unilateral nasal obstruction then instructed to alternatively close nostrils and indicate their more obstructed side. They were finally subject to nasal endoscopy, convexities in the nasal septum were noted, whether caudal septal deviation, anterior or superior septal deflection or posterior septal spur. Guruyon classification was followed.

### Tympanometry

Middle ear function can also be evaluated by impedance audiometry or tympanometry which renders specific graphs for ear pathologies [8]. Applying this tool in ET dysfunction is not yet well recognized systematically. A type C tympanogram reflects an ET dysfunction, which is a frequent pattern of subclinical middle ear malfunctions [8, 16]. Although a negative middle ear tube pressure on tympanometry, especially when the peak is below − 100 daPa, can indicate an ET dysfunction, the diagnosis remains presumably clinical.

### Statistical analysis

Statistical analyses were performed using Statistical Package for Social Science (SPSS) version 25 (IBM SPSS Software, Chicago, IL, USA). Descriptive statistics were calculated using mean and standard deviation for continuous measures, counts and percentages for categorical variables. The Kaiser–Meyer–Olkin (KMO) measure of sampling adequacy and Bartlett’s test of sphericity were ensured to be adequate. In the bivariate analysis, the Mann-Whitney test was used to compare the means of 2 groups, whereas the Spearman correlation coefficient was used to correlate between continuous variables. A *p* < 0.05 was considered statistically significant.

## Results

### Descriptive analysis

The mean age of the patients was 38.18 ± 14.72 years, with 51.7% females. Other characteristics of the patients can be found in Table 1. It is noteworthy that 5 (8.3%) patients had obstructive sleep apnea.

**Table 1:** Descriptive analysis of the participants (*N* = 60)

Variable	N (%)
**Gender**	
Male	29 (48.3%)
Female	31 (51.7%)
**Allergy**	
No	30 (50.0%)
Yes	30 (50.0%)
**Smoking**	
No	36 (60.0%)
Yes	24 (40.0%)
**Septal deviation**	
Right	33 (55.0%)
Left	27 (45.0%)
**Side symptoms**	
Right	31 (51.7%)
Left	29 (48.3%)
**Obstructive Sleep Apnea**	
No	55 (91.7%)
Yes	5 (8.3%)
	**Mean ± SD**
**Age (in years)**	38.18 ± 14.72
**Altitude (in meters)**	373.83 ± 353.05
**Right TPP (daPa)**	− 32.20 ± 86.38
**Left TPP (daPa)**	− 42.71 ± 87.24

### Bivariate analysis

A significantly higher ETD score was found in males compared to females (30.90 vs 26.19), in those with left septal deviation compared to right (30.41 vs 26.88) and in those who have symptoms on the left side compared to the right side (30.07 vs 29.97; *p* = 0.035). Frequent exposure to higher changes in altitude (commute from home to workplace) was also significantly associated with higher ETD scores (*r* = 0.265), whereas higher Left TPP (daPa) on tympanometry was significantly associated with lower ETD score (*r* = − 0.496) (Table 2).

**Table 2:** Bivariate analysis of factors associated with the ETD score

Variable	ETD score
**Gender**	
Male	30.90 ± 4.57
Female	26.19 ± 5.74
*p*	**0.003**
**Allergies**	
No	28.53 ± 5.96
Yes	28.11 ± 3.98
*p*	0.716
**Smoking**	
No	28.81 ± 5.94
Yes	27.96 ± 5.36
*p*	0.332
**Septal deviation**	
Right	26.88 ± 6.00
Left	30.41 ± 4.67
*p*	**0.03**
**Side symptoms**	
Right	29.97 ± 5.59
Left	30.07 ± 5.42
*p*	**0.035**
**Obstructive Sleep Apnea**	
No	28.73 ± 5.63
Yes	25.00 ± 4.53
*p*	0.153
	**Correlation coefficients**
**Age**	*r* = −0.045; *p* = 0.733
**Altitude**	*r* = 0.265; ***p*** **= 0.041**
**Right TPP (daPa)**	*r* = −0.176; *p* = 0.217
**Left TPP (daPa)**	*r* = −0.496; ***p*** **< 0.001**

Numbers in bold indicate significant *p*-values.

**Fig. 1:**
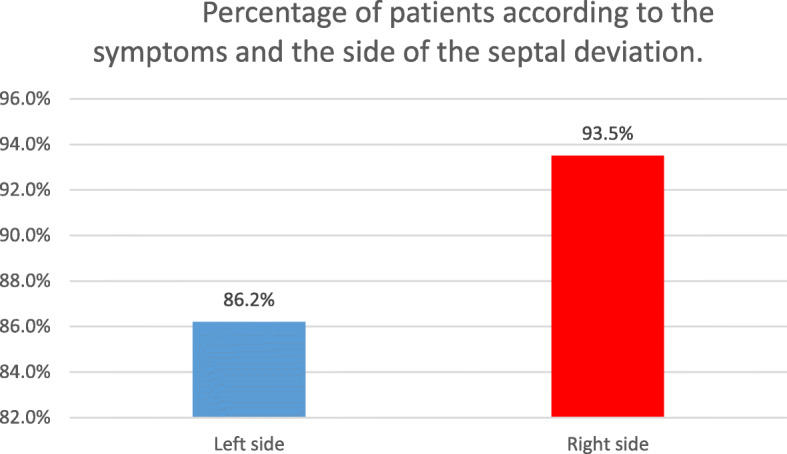
Percentage of patients according to the symptoms and the side if the septal deviation

Furthermore, 25 patients who had symptoms on the left side had also a septal deviation to the left side (86.2%), whereas 29 (93.5%) patients who had the symptoms on the right side had septal deviation to the right side (*p* < 0.001) (Fig. 1).

## Discussion

This cross-sectional study conducted on a sample of the Lebanese population is the first to determine the correlation between the side of reported symptoms of ETD and the side of NSD in patients suffering from the two conditions. We were able to assess the effect of altitude on the positivity of the ETDQ7, as well as to highlight the importance of the clinical assessment of the patient before any type of intervention. Our findings stated the frequency of ETD among men but was not conclusive regarding allergy, tobacco or tympanometry as a diagnostic tool.

### Demographics

In our study, most patients with a high ETD score were men. This finding mirrors others studies published in the literature such as Sereflican et al. [17]. Patel et al., studying this condition among children found more cases among boys as well [18]. Gallardo et al., on the other hand, noted a female predominance [3]. Akiyildiz et al. stated no difference regarding age or gender [15].

### Septal deviation

Many studies, such as those conducted by Kaya et al., Salvinelli et al., and Deron et al. showed that surgical correction of nasal septal deviation improves tubal opening pressure and affect middle ear ventilation [12, 19, 20]. However, none shed light on the impact of septal deviation on the ipsilateral Eustachian tube mechanism. Our study tried to address this point. We were interested in identifying any correlation between having worse symptoms related to Eustachian tube dysfunction on one side and presenting a septal deviation on the same side. Our findings state that 25 (86.2%) of the patients who had the symptoms on the left side presented with a septal deviation on the same left side, whereas 29 (93.5%) of the patients who had the symptoms on the right side had a septal deviation to the right side with great statistical significance (*p* < 0.001).

Thus, the side of the symptoms reported by patients with positive ETDQ-7 scores (above 14.5) suggest the side of the NSD with good accuracy. From our standpoint, any surgical intervention to the ear or the nose should be preceded by a diligent investigation of both structures, as we all pursue the best care for our patients, reflected first by symptom relief. We hypothesize that an underlying NSD, by obstructing airway passages may be a corresponding inducer to pressure differentiation and thus contribute to the pathophysiology of ETD in compromising middle ear ventilation.

In the recent medical literature, ETD is increasingly being managed with ET balloon dilation (with or without tympanostomy tube insertion) without always discussing or addressing the underlying nasal anatomy and condition [6]. However, some studies have looked at the impact of septoplasty on ET functions [15]. Akyildiz et al. concluded that septal surgery improved the ET function after conducting case-control studies on patients who underwent NSD surgical correction. Interestingly, they concluded that nasal surgeries may cause ET dysfunction soon after the procedure [15]. Sereflican et al. on the other hand, studied the effect of cannulated silicone intranasal splints, and concluded that their usage post septoplasty did not affect ET function [15].

### Altitude

A significant correlation was found between frequent exposure to high altitude variations and a high ETDQ-7. This finding mirrors that of previous medical literature on barotrauma [21]. No data has yet been established regarding frequent exposure to high altitude variation, but barotraumatic injuries have been closely monitored in pilots [21]. A previous study conducted on aviators in 2013, showed a pathological value of the ET score-7 after a hypobaric chamber session [21]. During ascent, middle ear pressure is higher than the external auditory canal’s pressure. This in turn pushes the tympanic membrane outward, thus forcing the ET to open and provide equal pressures [21]. However, during descent, the opposite happens [21]. This frequent exposure to pressure variability may induce changes in the motility of the tube. The geography of Lebanon, with close proximity of the mountains range to the main cities, and the narrow shoreline is somehow unique. Many patients commute every day from their home in the mountains to work on the coast. It is in the light of this topographical specificity that our results must be interpreted.

### Allergy

Upper airway infections and inflammation can induce some episodes of ET deregulation for example in conditions like allergic rhinitis, acute or chronic rhinosinusitis [4]. Many reports by Down et al. and others underlined the effect of histamine and aeroallergen in blocking the ET, suggesting that allergic rhinitis causes or aggravates this condition [16, 22]. Notifiable symptoms are exacerbated in allergy and pollen seasons [16]. Juszczak et al. in 2018 supported with evidence the connection between allergic rhinitis and ETD [16]. Previous findings by Hardy et al. on rats also provided greater insight on allergen induced inflammation and subsequent ETD [23]. Our study used the SFAR score in patients with positive ETDQ7 but we were not able to draw statistically significant conclusions correlating allergy to worse ETD Symptoms (*p* = 0.716).

### Tobacco

Conflicting findings exist regarding tobacco exposure and ET dysfunction [18]. Some authors have argued that perturbation of mucociliary clearance mechanisms and chemical irritation induced by smoking could contribute to ear discomfort but with no sufficient evidence [18]. Patel et al. found an association between passive smoking and ET dysfunction among children without any specific data regarding adults [18]. Our results have failed to achieve statistically significant correlation between ETD and tobacco exposure (*p* = 0.332).

### Evaluation of the Lebanese version of the ETDQ-7

The present study is the first to be conducted in Lebanon to evaluate the Arabic version of the ETDQ7. Our goal was to test this scale among a sample of the Lebanese population in the aim to improve patient diagnosis, management and follow up before and after interventions. Like any other quality of life questionnaire, it transformed the non-specific reported symptoms of the patients into a more objective, well standardized method of severity evaluation [3]. Regarding the relative prevalence of this disorder, around 5% in our population [3, 5] and considering the lack of a solid instrument to detect this entity, evaluating this scale was a necessity. Our country would benefit from a low-cost questionnaire due to the limited resources available [3]. As in the original study by Macoul et al. in 2012, and by Gallardo et al. in 2018, our patients did not receive any prior medical or surgical treatment [3].

The ETDQ-7 was already been used to discriminate between patients and non-patients with a set cut-off point of 14.5. It gained a sensitivity and specificity of 100 with Macoul et al., and 95 and 97% respectively with the Brazilian version of the scale [3, 5]. The previous studies were cross sectional studies comparing two groups: patients and non-patients. Our study, based on patients only, demonstrated a Cronbach alpha coefficient of 0.409 compared to 0.746 in the original study by Macoul et al., and the Brazilian study. None of the items of ETD scale was removed. An additional case-control study validating the Turkish version of the scale registered a Cronbach alpha coefficient of 0.714 for the whole scale in the test-retest reliability, close to the 0,746 obtained with previous studies [9]. An important review published by Teixeira et al., tested the accuracy of this scale, and concluded that even if the scale correlated with patients’ symptoms, it did not relate with an objective measure of ET function [14]. The test used was the percentage of middle ear pressure equalization after 5 swallows (PEq5) However, our data showed that this scale may be significantly suggestive of patients’ symptoms. Our study aimed to see if the cut-off score of 14.5 already used in literature is applicable in our sample.

A study conducted by Alper et al. showed that tubomanometry cannot be used to evaluate or diagnose the ET function [7]. However, tubomanometry was used by Ockermann et al. to develop a Eustachian Tube Score: it consisted of three tubomanometry measurements with two patient assessments after performing both Valsalva and Toynbee manoeuvers [21]. Tympanometry is used in a limited number of studies including the National Health and Nutrition Examination Survey (NHANES) study 2005–2010 where it was considered the main useful tool in diagnosing ETD [18]. Gallardo et al. in 2016, also concluded that a normal tympanogram ruled out the disease [3]. Another study conducted in India considered a type-C tympanogram < − 100 daPa as evidence of ETD [22].

The study we conducted is a cross-sectional study. As shown in Table 2, higher Left TPP was significantly associated with lower ETD score with *p* < 0.001 in our population. Left TPP was dispersed around − 42.71 ± 87.24. So, a non-type C tympanometry did not rule out ETD based on the cut-off set for ETDQ-7. In many instances, ETDQ-7 score was still above 14.5 even in patients with TPP > − 100 daPa. Our results only suggest that when left TPP increases, the ETDQ7 score decreases 0.496. This finding sheds a doubt on the reliability of tympanometry as a screening tool for ET Dysfunction in our population and raises uncertainty on how and where to apply this tool. These results need to be interpreted in the light of the previously discussed specificity of our study population in regards to frequent exposure to altitude variation. Our patients could be suffering more from baro-traumatic, transient or fluctuating ET Dysfunction. This could explain normal findings on tympanometry results at the time of the test conduction in a facility located at sea level. Future larger studies will be needed to confirm these findings.

### Limitations

Inherent limitations to our study include being a single centre study, based on patients’ self-reported symptoms. Others studies have looked at surgical interventions to recruit their control group, studying mainly the effect of these interventions on the ET function and evaluating at the same time the ETDQ7 questionnaire. While the study attempts to evaluate the scale, it must be recognized that the analysis is bounded by a limited number of patients. Perhaps one of the biggest limitations to our study is in the studied population itself, as our study group lives in a country with frequent, if not daily exposure to large variations of altitude, thus maybe describing a specific pattern or pathophysiology of ETD related to barotrauma. Opportunities for future studies with larger samples should be considered.

## Conclusion

This study has looked for the first time at the correlation between the asymmetric ETD and ipsilateral NSD, shedding the light on an often-overlooked clinical observation. Our data also highlighted the importance of altitude and geographic distribution of patients especially in a population exposed to barotrauma on a daily basis like the Lebanese population. The ETDQ7 questionnaire is of great value for evaluating this dysfunction. Tympanometry, on the other hand, failed to correlate with patient reported symptoms and thus needs further evaluation.

## Data Availability

The authors do not have the right to share any data information as per their institutions policies.
